# Uninephrectomy in rats on a fixed food intake results in adipose tissue lipolysis implicating spleen cytokines

**DOI:** 10.3389/fphys.2015.00195

**Published:** 2015-07-10

**Authors:** Denis Arsenijevic, Jean-François Cajot, Abdul G. Dulloo, Jean-Pierre Montani

**Affiliations:** ^1^Division of Physiology, Department of Medicine, University of FribourgFribourg, Switzerland; ^2^National Center of Competence in Research Kidney.CHZurich, Switzerland

**Keywords:** uninephrectomy, lipolysis, body composition, cytokines, spleen

## Abstract

The role of mild kidney dysfunction in altering lipid metabolism and promoting inflammation was investigated in uninephrectomized rats (UniNX) compared to Sham-operated controls rats. The impact of UniNX was studied 1, 2, and 4 weeks after UniNX under mild food restriction at 90% of *ad libitum* intake to ensure the same caloric intake in both groups. UniNX resulted in the reduction of fat pad weight. UniNX was associated with increased circulating levels of beta-hydroxybutyrate and glycerol, as well as increased fat pad mRNA of hormone sensitive lipase and adipose triglyceride lipase, suggesting enhanced lipolysis. No decrease in fat pad lipogenesis as assessed by fatty acid synthase activity was observed. Circulating hormones known to regulate lipolysis such as leptin, T3, ghrelin, insulin, corticosterone, angiotensin 1, and angiotensin 2 were not different between the two groups. In contrast, a select group of circulating lipolytic cytokines, including interferon-gamma and granulocyte macrophage–colony stimulating factor, were increased after UniNX. These cytokine levels were elevated in the spleen, but decreased in the kidney, liver, and fat pads. This could be explained by anti-inflammatory factors SIRT1, a member of the sirtuins, and the farnesoid x receptor (FXR), which were decreased in the spleen but elevated in the kidney, liver, and fat pads (inguinal and epididymal). Our study suggests that UniNX induces adipose tissue lipolysis in response to increased levels of a subset of lipolytic cytokines of splenic origin.

## Introduction

Disease conditions such as the metabolic syndrome, diabetes, obesity, inflammation and infection, are often associated with diminished kidney function. It is generally believed that this reduction in kidney function is a consequence of the progression of the disease. Recent evidence in both humans and animal models suggests that a primary reduction in kidney function may also play a role in altering metabolism (Odamaki et al., 1999; Zhao et al., 2011) inflammation and oxidative stress (Zheng et al., 2011) and hence in the pathogenesis of the disease.

Previous studies of the consequences of uninephrectomy (UniNX) in Sprague Dawley rats have shown that there was no difference in body weight and no evident changes in metabolic profile and tissue pathology up to 3 months. Afterwards, pathologies start to appear, in particular deterioration of kidney function, fatty infiltration into various tissues (Zhao et al., 2008, 2011) and the progressive development of glucose intolerance (Sui et al., 2007). However, the mechanisms underlying these temporal changes from subtle changes to chronic severe changes in metabolic and immune regulation are not clearly defined. Angiotensin may play a role in metabolic and immune changes observed in kidney disease (Amorena et al., 2001; Deferrari et al., 2002), but its contribution in early reduced kidney function remains to be determined.

More recently, studies have shown that other factors regulating metabolism and inflammation are modified by diminished kidney function in humans (Wu et al., 2006; Spoto et al., 2012) and in rodent UniNX models (Gai et al., 2014b), including the sirtuin SIRT1, farnesoid X receptor (FXR), inflammation and complement factors. Activation of SIRT1 and FXR can counter the metabolic syndrome by acting on lipid and glucose metabolism. We and others have recently shown that bile salts and their receptor FXR are modified by UniNX (Penno et al., 2013; Gai et al., 2014a,b; Chin et al., 2015). It has also been reported that SIRT1 may regulate FXR activity (Liu et al., 2014).

Only a few studies have investigated the role of cytokines in UniNX-induced metabolic changes (Mak et al., 2006; Zhang et al., 2014). However, recent studies in mice suggest that cytokines and their signaling pathways are altered by UniNX (Zheng et al., 2011; Gai et al., 2014b). In a more severe form of reduced kidney function, 5/6 nephrectomy, cytokines have been shown to play a role in pathology (Gao et al., 2011). The role of cytokines in metabolic disease especially concerning lipid metabolism is complex as the dose administered of the cytokine is important; at different doses different phenotypes can occur (Feingold et al., 1992; Khovidhunkit et al., 2004). Furthermore, the source of cytokines in kidney disease (Spoto et al., 2012) may not be the same as in obesity or metabolic disease where adipose tissues are believed to be a major source (Fruhbeck et al., 2001). In other inflammation/infection models, other tissues such as spleen and liver can be a major source of cytokines (Arsenijevic et al., 1998; Park et al., 2010).

It has been shown in chronic human kidney disease that there is an association between circulating cytokines and body weight (Pecoits-Filho et al., 2002). At both extremes of body weight perturbations, obesity and cachexia, it has been shown that cytokines can alter body composition and metabolic pathways. These pathways include protein, lipid and glucose metabolism (Johnson, 1997). Cytokines can act directly on tissue or indirectly via the brain to affect tissue metabolism (Johnson, 1997; Sanchez-Lasheras et al., 2010).

In pilot studies we found that UniNX decreased fat pad weight and increased certain circulating cytokines. We therefore conducted studies to investigate whether UniNX induces changes in body composition, in particular body fat pad lipolysis and lipogenesis, under conditions of fixed food intake (90% of *ad libitum* intake) and whether those changes are associated with selected hormones or cytokines. We also investigated the tissue source of lipolytic cytokines and whether anti-inflammatory tissue regulators FXR/SIRT1 were modified in tissues.

## Methods

### Animal preparation and experimental protocol

#### Animal model

Male Sprague Dawley rats were purchased from Elevage Janvier (Le Genest-St-Isle, France) at 5 weeks of age with an average weight of 160 g/rat. They were placed individually in cages and given pellet food *ad libitum*. After a 1 week acclimation period, rats were either sham operated or uninephrectomized (UniNX) by removal of the left kidney. One day prior to surgery a group of eight non operated rats were sacrificed (day 0 group).

#### Surgery

Rats were first anesthetized with sevoflurane and then placed on a heated mat. The left flank was shaved and swabbed with polyvidone-iodine (Braunoderm, Braun). Anesthesia and analgesics were given i.p.: medetomidine hydrochloride (Domitor) 150 μg/kg, Midazolam (Dormicum) 2 mg/kg, fentanyl 5 μg/kg and to awake by atipamezole hydrochloride (Antisedan) 0.75 mg/kg, Sarmazenil (Sarmasol) 0.2 mg/kg, Naloxone (Narcan) 120 μg/kg. A small incision was made in the left flank to gain access to the left kidney. The kidney was ligated with non-absorbable thread (Ethilon 11 4-0, Johnson–Johnson) and was then cut loose with surgical scissors. The incision sites were sutured with absorbable thread (Vicryl 3-0 Johnson–Johnson) and metal Michel suture clips (Provet, Switzerland) were applied to close the wound. Metal clips were removed after 14 days. Post-operation analgesic treatment with buprenorphine 0.05 mg/kg s.c. was given 2X/day for 3 days to Sham and UniNX animals.

#### Diet

Most of the studies analyzing the impact of UniNX have been done under *ad libitum* fed conditions. We chose instead to put the rats under a fixed food intake (90% of ad lib fed diet) to ensure the same caloric intake between sham and UniNX rats. *Ad lib* feeding results in uncontrolled levels of nutrition, which can influence metabolites, hormones, inflammation and oxidative stress, parameters of interest (Diamond, 1990). Dietary intake differences can also influence other variables such as locomotor activity and metabolic rate (Leveille and O'Hea, 1967). Therefore, fixed intake obviates some of these confounding factors encountered in *ad libitum* experiments. This fixed intake approach has previously been used successfully to study the mechanisms underlying body composition regulation during catch-up growth and energy balance in young Sprague–Dawley male rats (Summermatter et al., 2009). After surgery animals were given a fixed intake of normal chow paste. Dry food powder (Maintenance diet composed of 23.5% protein, 12.9% fat, and 63.6% carbohydrates as percentage of metabolisable energy: Cat. No. 3433, Provimi-Kliba, Cossonay, Switzerland) was mixed with an equal amount of tap water and was prepared daily (equivalent to 90 kcal/rat) and given in food cups.

#### Experimental protocol

Rats were kept in individual cages and had free access to water. The environmental temperature was maintained at 22 ± 1°C in a room with a 12 h light/dark cycle (light 7.00 a.m.–7.00 p.m.). Body weight was measured daily before feeding (9.00–11.00 a.m.). Operated rats were sacrificed at 1, 2, and 4 weeks after surgery. At each time point, eight sham rats and eight UniNX rats were sacrificed for collection of blood, tissue samples and animal carcasses. Rats were anesthetized with ketamine (70 mg/kg) for sacrifice, then decapitated for immediate blood collection. Animals were placed on ice for collection of peritoneal macrophage using pyrogen free phosphate saline buffer (see below) and small pieces of tissues were collected for analysis. All experimental protocols were approved by the Ethical Committee of the Veterinary Office of Fribourg, Switzerland.

### Body composition

For body composition analysis the rats were killed by decapitation. The skull, thorax and abdominal cavity were incised and the gut was cleaned of undigested food. The carcasses were dried in an oven maintained at 60°C for 2 weeks, after which they were homogenized. Carcass fat content was measured by the Soxhlet fat extraction method using petroleum-ether (Entenman, 1957). Body water content was determined by subtracting the weight of the animal after the 2 weeks in the oven to the weight prior to this. The fat free dry mass (FFDM) was calculated as the fat mass subtracted from the dry homogenate.

### Blood parameters

Blood was collected on ice (in EDTA- or heparin-coated tubes) and centrifuged at 4°C at 3000 rpm in a microcentrifuge. Serum and plasma were then kept at -20°C until analyzed. For a complete list of metabolites, hormones analyzed and the provenance of kits, see Table 1.

**Table 1:** **Assay kits for metabolites, hormones, and cytokines**.

**Assay kit**	**Kit name/Cat. No**.	**Company**
**METABOLITE ASSAY KITS FOR USE ON SYSTEM ROCHE/HITACHI ANALYSER COBAS C501**		
Urea	UREAL kit	1
Triglycerides	TRGL kit	1
Cholesterol	Total cholesterol CHOL2 kit	1
HDL	High density lipoprotein HDLC3 kit	1
**METABOLITE PLATE ASSAY KITS**		
Free fatty acid assay kit	Cat. No. K612-100	2
β-Hydroxybutyrate assay kit	Cat. No. K623-100	2
Glycerol assay kit	Cat. No. K630-100	2
**HORMONE ELISA OR EIA**		
Aldosterone	EIA kit Cat. No. 10034377	3
Leptin	EIA kit for Mouse/rat Cat. No. A05176	3
Corticosterone	EIA kit Cat. No. 5006553	3
Ghrelin	EIA kit Cat. No. EK-031-31	4
Angiotensin-1	EIA kit Cat. No. EKE-002-01	4
Angiotensin-2	EIA kit Cat. No. EK-002-12	4
T3 total	ELISA kit Cat. No.90060	5
Insulin	EIA kit Cat. No. 07BC1005	6
**CYTOKINE ELISA KITS AND OTHER KITS**		
Erythropoietin	ELISA Cat. No. DY959	7
Interleukin(IL)1α	ELISA Cat. No. DY500	7
IL1β	ELISA Cat. No. RLB00	7
IL1RA	ELISA Cat. No. DY480	7
Granulocyte-Macrophage Colony Stimulating Factor (GM-CSF)	ELISA Cat. No. DY518	7
C-reactive protein (CRP)	ELISA Cat. No. DY1744	7
IL4	ELISA Cat. No. BMS628MST	8
IL6	ELISA Cat. No. BMS625	8
IL10	ELISA Cat. No. BMS629	8
Tumor Necrosis factor (TNFα)	ELISA Cat. No. 88-7340	8
Interferon-gamma (IFNγ)	ELISA Cat. No. BMS621	8
Acylation Stimulating Protein (ASP)	ELISA Cat. No.MBS728340	9
Serum Neopterin	ELISA Cat. No. RE59321	10
Cystatin-C	Immunoassay Cat. No. KK-CYC	11

1, Roche, Switzerland; 2, Biovision, Milpitas, CA, 95035, USA; 3, Cayman Ann Arbor, Michigan, 48108, USA; 4, Phoenix Europe, D-76133, Karlsruhe, Germany; 5, Crystal Chem, IL, USA; 6, MP Biomedicals Europe, Illkirch, 67402, France; 7, R&D, Abingdon OX14 3NB, UK; 8, eBioscience – San Diego, CA 92121, USA; 9, MyBiosource – San Diego, CA 92195, USA; 10, IBL Toronto, ON, M3J 2N5, Canada; 11, Buhlmann Basel Switzerland.

### RT-PCR in epididymal/inguinal white adipose tissue (EWAT/IWAT) and liver

Total RNA was isolated as previously described (Arsenijevic et al., 1997). The RNA was then treated with DNase, after which it was reverse transcribed (Promega). Thereafter we ran a RT-PCR (iQ cycler Bio-Rad). Each sample was normalized to its cyclophilin value. For the list of primers used and their sources, see Table 2. Samples were incubated in the iCycler instrument (BioRad, iCycler iQ, Version 3.1.7050) for an initial denaturation at 95°C for 3 min, followed by 40 cycles of amplification. Each cycle consisted of 95°C for 10 s, 60 or 62°C for 45 s, and finally 95, 55, and 95°C for 1 min each. Green I fluorescence emission was determined after each cycle. The relative amount of each mRNA was quantified by using the iCycler software. Amplification of specific transcripts was confirmed by melting curve profiles generated at the end of each run. Cyclophilin was used as the control for each study and the relative quantification for a given gene was normalized to cyclophilin mRNA values. Note that as representative of subcutaneous white adipose tissue (SWAT) we used inguinal fat (IWAT) for PCR, western blot and other analysis.

**Table 2:** **RT-PCR primers**.

**Primers name, sequence, and original source of sequences**
Adipose triglyceride lipase (**ATGL)**sense 5-TGTGGCCTCATTCCTCCTAC-3, antisense 5-AGCCCTGTTTGCACATCTCT-3 (Palou et al., 2009)
Hormone sensitive lipase (**HSL)**sense 5-TCACGCTACATAAAGGCTGCT-3, antisense 5-AGTTCCCTCTTTACGGGTGG-3 (Palou et al., 2009)
**CD36** sense 5-GTCCTGCCTGTGTGA-3, antisense 5-GCTCAAAGATGCTCCATTG-3 (Palou et al., 2009)
**Cyclophilin** sense 5-TCAGGGCTCTTGAAGTCCC-3, antisense 5-CAGAAAATCACAGCAGCCAAC-3 as reference control (Summermatter et al., 2009)

### Western blot analysis

Western blots on protein extracts from pulverized tissue were performed as previously described (De Bilbao et al., 2009). Protein samples were loaded at 30 μg/20 μl, after migration proteins were transferred by semi-dry transfer (De Bilbao et al., 2009). Membranes were pre-incubated with 1% casein (Vectorlab), then incubated 2 h with primary antibody uncoupling protein-1 (UCP1) dilution 1/5000 (cat. no. UCP11, Alpha Diagnostics), SIRT1 dilution 1/200 (sc-19857, Santa Cruz), FXR 1/200 dilution (sc-13063, Santa Cruz), and beta-actin dilution 1/1000 (Cat No. 4970—Cell Signaling). Secondary antibody LI-COR anti-rabbit (1/15000) or anti-goat (1/15000) were used to detect bands (De Bilbao et al., 2009). The signals were visualized with the use of Odyssey Infrared Imaging System (LI-COR Biosciences, Bad Homburg, Germany).

### Lipogenic enzyme activity assays

Fatty acid synthase (FAS) activity was measured according to a method described by Penicaud et al. (1991). The frozen white adipose tissue pads were homogenized on ice in four volumes of freshly prepared polyethylene glycol buffer, pH 7.3 (100 mmol/l KH_2_PO_4_, 5 mmol/l EDTA, and 1.5 mg/ml glutathione in reduced form). After centrifugation, these extracts were assayed using 15 μl of extract in 1.7 ml of FAS buffer (50 mmol/l K-phosphate stock solution, pH 6.8, and 0.1 mg/ml NADPH) and using a spectrophotometer set at 340 nm and 37°C. The readings were performed by sequentially adding 15 μl of extract in 1.7 ml of FAS buffer to the cuvettes, followed by 10 μl of 7.5 mmol/l acetyl-CoA, and followed by 10 μl of 8 mg/ml malonyl-CoA.

### Circulating cytokines and markers of immune activation

Rat serum ELISA assays for interleukin (IL)1α, IL1β, IL1Receptor Antagonist (IL1RA), erythropoietin (EPO) and C-reactive protein (CRP) were purchased from R&D, Abingdon, OX14 3NB, UK. Other cytokine ELISA kits were purchased from eBioscience, San Diego, CA 92121, USA, including IL4, IL6, IL10, tumour necrosis factor alpha (TNFα), interferon-gamma (IFNγ) and granulocyte macrophage colony stimulating factor (GM-CSF). Acylation stimulating protein (ASP) was measured by ELISA from MyBiosource. Serum Neopterin, a by-product specific of IFNγ activated macrophages, was also determined by ELISA (IBL Toronto, ON, M3J 2N5, Canada). For a complete list of cytokines and provenance see Table 1.

### Cytokine levels in tissues

Tissue cytokine determination was performed, as previously described (Arsenijevic et al., 2006), on tissues from week 4 post UniNX. Briefly, 100 mg of tissue were homogenized with 600 μl of 1% CHAPS (3-[(3-cholamidopropyl) dimethylammonio]-1-propanesulfonate) in RPMI-1640 medium without Phenol red (R7509, Gibco) with a polytron homogenizer (Nakane et al., 1992). The supernatant was collected and frozen at −20°C (Arsenijevic et al., 2006). Cytokines mentioned above were assayed using immunoassay kits, as described previously (Arsenijevic et al., 2006).

### Macrophage intracellular ROS production

Reactive oxygen species (ROS) production was measured from isolated macrophages by measuring their ability to reduce nitro blue tetrazolium. Peritoneal macrophage layers in 96 well plates were isolated from peritoneal cavity of Sham and UniNX (*n* = 8) with ice cold pyrogen free phosphate buffered saline (PBS). After being centrifuged and washed with PBS three times macrophages were counted and plated at 100,000 per well and let to adhere to plates by incubating at 37°C for 30 min. After this period a solution of nitro blue tetrazolium with 5% glucose in PBS was incubated for a further 3 h at 37°C. The supernatant was removed and gently washed with PBS 3 times. Cells were then fixed with 70% methanol and allowed to dry. Formazan was solubilized with 2 M KOH and dimethyl sulphoxide. The absorbance was determined at 630 nm (Arsenijevic et al., 2000).

### Data analysis

All data are presented as means ± SE. Statistical analysis were performed using Kruskal–Wallis One-Way non-parametrical ANOVA or Mann-Whitney (non-parametrical) for 2 sample comparisons. A value of *p* < 0.05 was considered as significant. ^*^p < 0.05, ^**^p < 0.01 and ^***^p < 0.001.

## Results

### Uninephrectomy effect on kidney function

**Figure 1:**
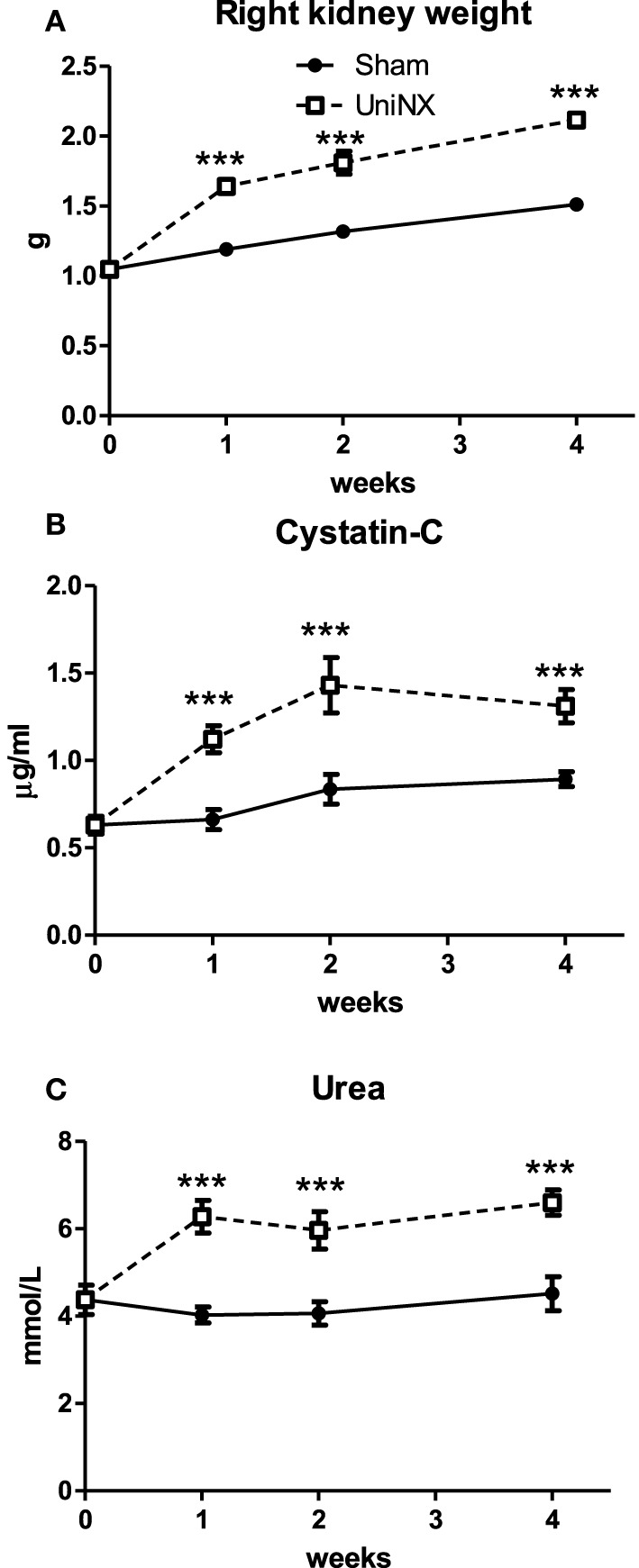
**Evolution over 4 weeks of the weight of the remaining right kidney of UniNX and of the right kidney of Sham animals (A)**. Evolution of plasma indicators of kidney function Cystatin-C **(B)** and Urea **(C)**. Values are means ± SE; *n* = 8/group. ^***^P < 0.001 corresponds to Sham vs. UniNX.

Left nephrectomy resulted in hypertrophy of the remaining right kidney (Figure 1A), which was 38% heavier than the right Sham kidney on week 4. A mild reduction in kidney function is reflected by the increased plasma Cystatin-C and urea levels (Figures 1B,C).

### Body weight, body composition, and organ weight

**Figure 2:**
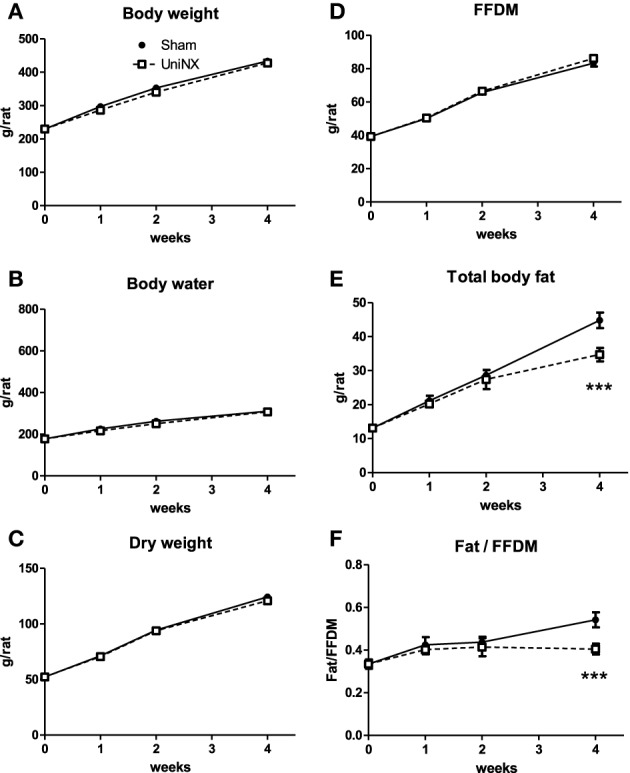
**Evolution over 4 weeks of body weight (A), body water (B), dry weight (C), total body fat (D), fat free dry mass–FFDM (E), and the ratio of body fat/FFDM (F) in Sham operated controls and UniNX rats**. Values are means ± SE; *n* = 8/group. ^***^P < 0.001 corresponds to Sham vs. UniNX.

**Figure 3:**
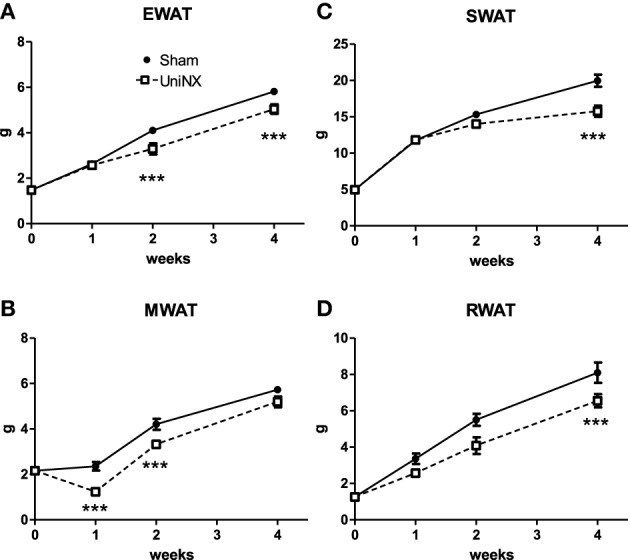
**Evolution over 4 weeks of fat pad weight, epididymal–EWAT (A), mesenteric–MWAT (B), subcutaneous–SWAT (C), and retroperitoneal–RWAT (D) in Sham operated and UniNX rats**. Values are means ± SE; *n* = 8/group. ^***^P < 0.001 corresponds to Sham vs. UniNX.

Over the 4 week period, there was no significant difference in body weight between the UniNX and Sham groups (Figure 2A). However, UniNX animals had a tendency to weigh less than Sham animals. Body composition analysis showed that body water, dry body weight and FFDM (Figures 2B–D) did not differ significantly between the two groups over the 4 week period. Total body fat was only reduced significantly by week 4 in the UniNX group (Figure 2E), which was reflected in the fat to FFDM ratio (Figure 2F). Fat pad weights were significantly decreased during the course of the 4 weeks. In general, the UniNX group had significantly lower epididymal, mesenteric, subcutaneous and retroperitoneal fat pads than Sham counterparts (Figures 3A–D). These significant decreases in fat mass were not associated with a significant increase in FFDM although there was a tendency for FFDM to be higher in the UniNX rats. This small increase may be explained by increases in non-muscle tissues such as spleen (Sham 1.12 ± 0.14 g/rat and UniNX 1.44 ± 0.15 *p* < 0.05) and gastrointestinal tract (for intestines—Sham 7.25 ± 0.83 g/rat vs. UniNX 8.50 ± 0.76 g/rat *p* < 0.01; stomach—Sham 1.52 ± 0.17 g/rat and UniNX 1.82 ± 0.17 g/rat *p* < 0.01). No significant differences between Sham and UniNX were seen for the liver weights.

### Blood lipid metabolites

**Figure 4:**
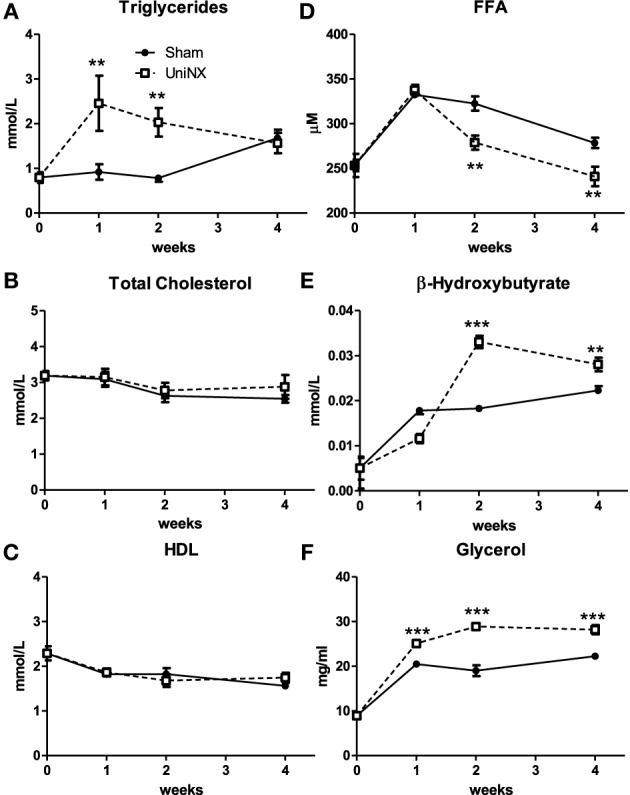
**Evolution over 4 weeks of triglycerides (A), total cholesterol (B), high density lipoprotein–HDL (C), free fat acids–FFA (D), β-hydroxybutyrate (E), and glycerol (F) in Sham operated and UniNX rats**. Values are means ± SE; *n* = 8/group. ^**^P < 0.01. ^***^P < 0.001 corresponds to Sham vs. UniNX.

Plasma triglyceride concentrations showed a transient increase after UniNX, declining after 1 week so that by week 4 UniNX triglycerides were similar to Sham levels (Figure 4A). Total blood cholesterol and high density lipoprotein (HDL) levels were not significantly different between Sham and UniNX groups (Figures 4B,C). However, from week 2 to week 4, free fatty acids in the UniNX group were reduced compared to the Sham group (Figure 4D). Blood β-hydroxybutyrate, a product of fatty acid oxidation, showed a marked increase from week 2 to week 4 (Figure 4E). Circulating glycerol, a product of lipolysis, was persistently elevated over the 4 weeks (Figure 4F).

### Lipid metabolism assessment by RT-PCR and western blots in tissues

**Figure 5:**
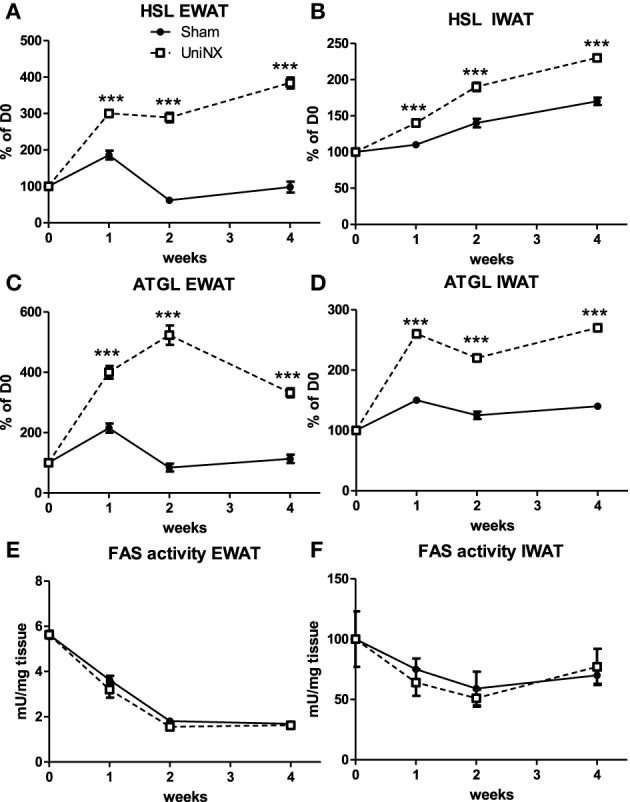
**Evolution over 4 weeks of mRNA levels of hormone sensitive lipase–HSL, adipose triglyceride lipase–ATGL, and fatty acid synthase–FAS activity in epididymal fat pad–EWAT (A,C,E) and inguinal fat pad–IWAT (B,D,F) in Sham operated and UniNX rats**. Values are means ± SE; *n* = 8/group. ^***^P < 0.001 corresponds to Sham vs. UniNX.

**Figure 6:**
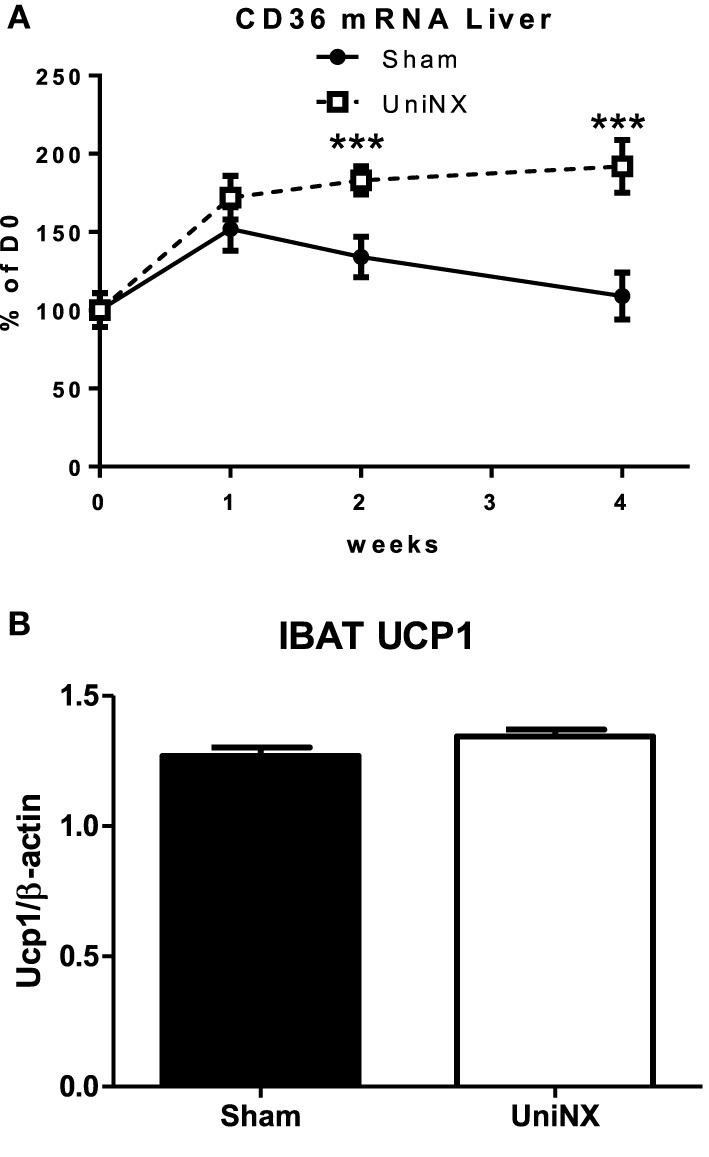
**Evolution over 4 weeks of liver free fatty acid transporter CD36 mRNA in Sham operated and UniNX rats (A)**. Interscapular brown adipose tissue (IBAT) UCP1 protein levels from western blot on the 4th week in Sham operated and UniNX rats **(B)**. Values are means ± SE; *n* = 8/group.^***^P < 0.001 corresponds to Sham vs. UniNX.

Hormone sensitive lipase (HSL) and adipose triglyceride lipase (ATGL) mRNA (Figures 5A–D) were elevated in the EWAT and IWAT fat pads over the 4 weeks in the UniNX group. Fatty acid synthesis as determined by FAS activities were similar in Sham and UniNX groups (Figures 5E,F) in EWAT and IWAT fat pads. The free fatty acid transporter CD36 mRNA in the liver was higher from week 2 to week 4 in UniNX animals than Sham (Figure 6A). In addition, we also observed increased CD36 mRNA in selected UniNX tissues (by 142% in the kidney and by 79% in the gastrocnemius muscle). Interscapular brown adipose tissue (IBAT) thermogenic uncoupling protein 1 (UCP1 protein levels) showed no differences between Sham and UniNX (Figure 6B) at 4 weeks.

### Blood hormones

**Figure 7:**
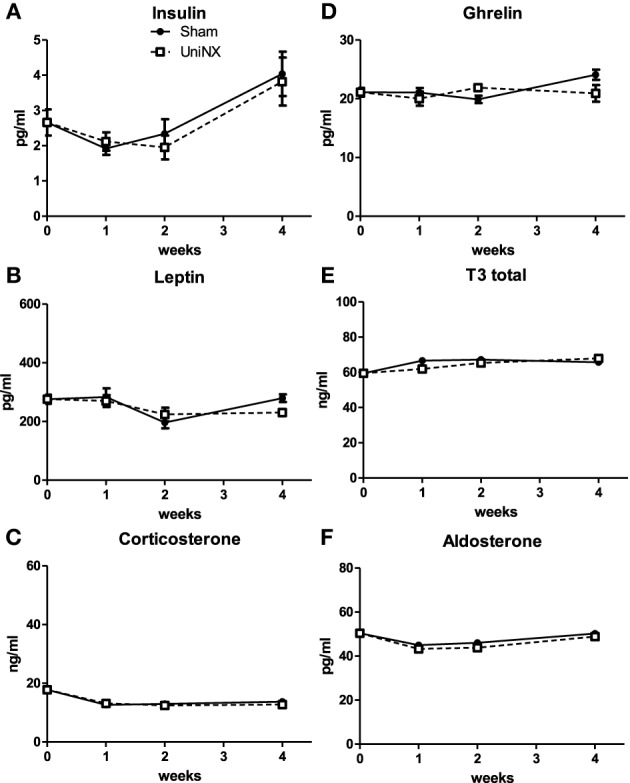
**Evolution over 4 weeks of plasma insulin (A), leptin (B), corticosterone (C), ghrelin (D), total T3 (E), and aldosterone (F) in Sham operated and UniNX rats**. Values are means ± SE; *n* = 8/group.

Blood hormones insulin, leptin, corticosterone, ghrelin, T3, and aldosterone (Figures 7A–F) were not significantly different between Sham and UniNX animals over the 4 weeks. We also observed that on week 4, UniNX and Sham groups showed similar levels of circulating angiotensin 1 (Sham: 4.4 ± 0.2 ng/ml and UniNX: 4.2 ± 0.2 ng/ml) and angiotensin 2 (Sham: 6.5 ± 0.3 ng/ml and UniNX: 6.2 ± 0.2 ng/ml).

### Serum and tissue cytokines

**Figure 8:**
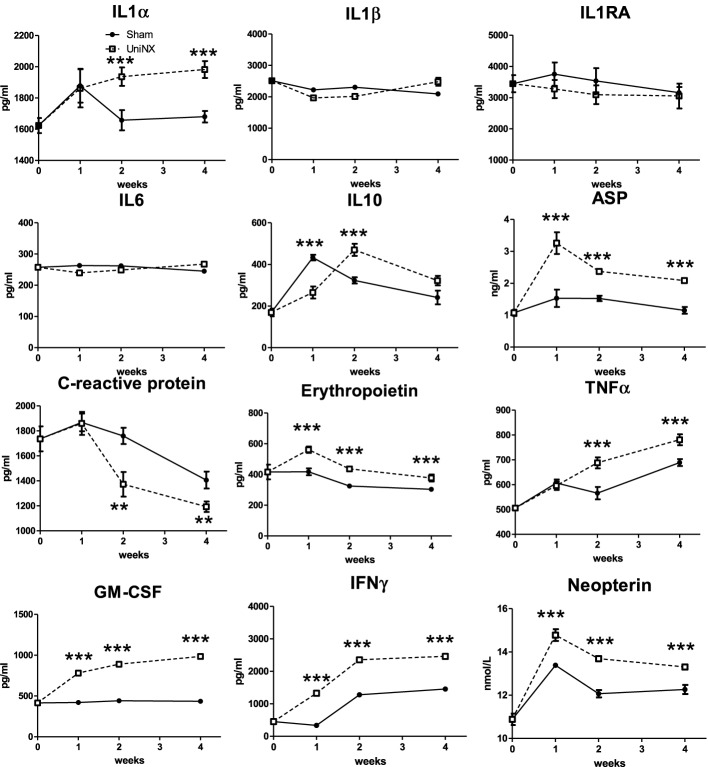
**Evolution over 4 weeks of serum IL1α, IL1β, IL1RA, IL6, IL10, ASP, C-reactive protein, erythropoietin, TNFα, GM-CSF, IFNγ, and Neopterin in Sham operated and UniNX rats**. Values are means ± SE; *n* = 8/group. ^**^P<0.01, ^***^P < 0.001 corresponds to Sham vs. UniNX.

IL1α, IL1β, IL1RA, IL6, IL10, ASP, CRP, EPO, TNFα, GM-CSF, IFNγ, and neopterin were measured in serum after UniNX. Serum IL1α, GM-CSF, EPO, IFNγ, and ASP were all higher in UniNX than in Sham controls from week 1 to week 4 (Figure 8). Neopterin, a specific indicator of IFNγ activated macrophages, was also higher over the 4 week period in the UniNX group than in Sham controls. CRP, an indicator of liver inflammation state, was lower in the UniNX group from week 2 to week 4 (Figure 8).

**Figure 9:**
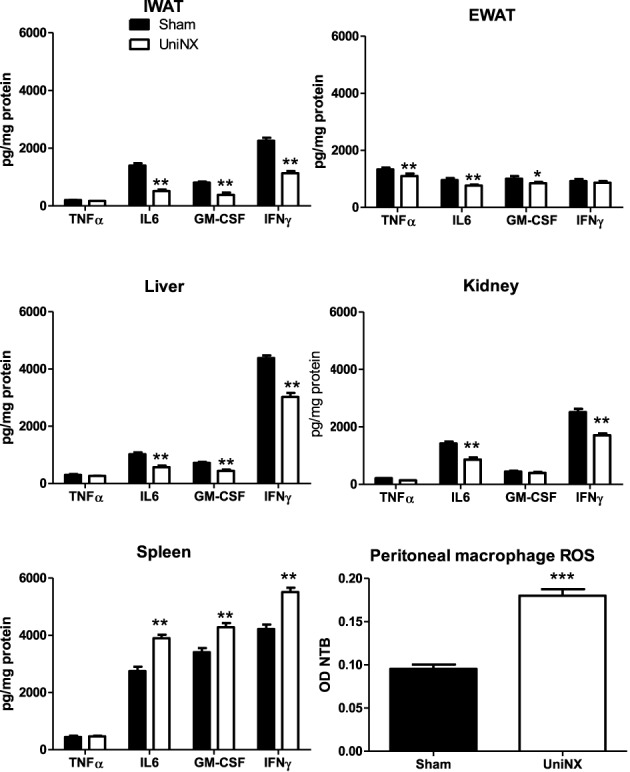
**TNFα, IL6, GM-CSF, and IFNγ cytokine levels in inguinal fat pad–IWAT, epididymal fat pad–EWAT, kidney, liver, and spleen on the 4th week in Sham operated and UniNX rats**. Peritoneal macrophage reactive oxygen production capacity as determined by measurement of nitro blue tetrazolium. Values are means ± SE; *n* = 8/group. ^*^P < 0.05, ^**^P < 0.01, ^***^P < 0.001 corresponds to Sham vs. UniNX.

Four selected cytokines (TNFα, IL6, GM-CSF, and IFNγ) were measured in various tissues at week 4, as shown in Figure 9. In most tissues, UniNX decreased tissue cytokine protein levels compared to the Sham group. The only tested UniNX tissue that showed a marked increase in the cytokines was the spleen. Peritoneal macrophage ROS production was doubled in UniNX rats (Figure 9), reflecting immune activation.

### Tissue SIRT1 and FXR protein levels

**Figure 10:**
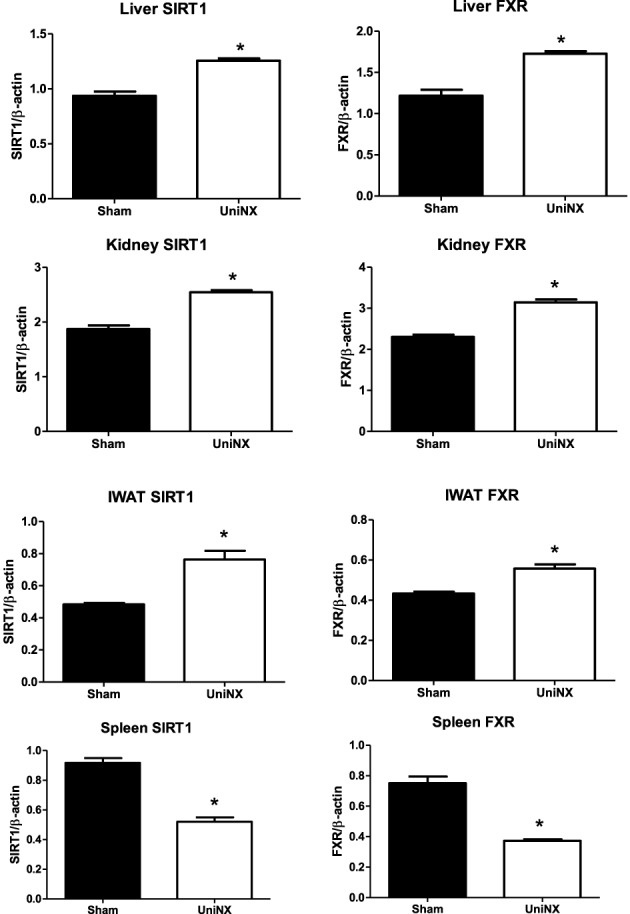
**SIRT1 and FXR protein levels in liver, kidney, IWAT, kidney, and spleen on the 4th week in Sham operated and UniNX rats**. Values are means ± SE; *n* = 8/group.^*^P < 0.01 corresponds to Sham vs. UniNX.

Since SIRT1/FXR have anti-inflammatory properties it was decided to determine whether their levels were modified by UniNX. On week 4 SIRT1 and FXR proteins levels in IWAT, EWAT, kidney, and liver were higher in UniNX animals than in Sham controls. In sharp contrast, SIRT1 and FXR were lower in UniNX spleen (Figure 10).

## Discussion

Compared to Sham controls, UniNX in young male rats resulted in a mild reduction in kidney function as judged by the chronic elevation of circulating Cystatin-C and urea over a 4 week period. No significant differences in body weight were observed between Sham and UniNX groups. However, UniNX reduced fat pad weight, and this decrease was also evident in total body fat content as determined by body composition analysis 4 weeks post UniNX. The causes of decreased fat pad weight could not be attributed to differences in food intake since we used fixed intake feeding; in addition it could not be explained by reduced FAS as no difference in the activity of this enzyme was found between the two groups in inguinal and epididymal fat pads. Since UCP1 protein levels were not different between the two groups, increased brown adipose tissue thermogenesis does not appear to be involved in the lower body fat content following UniNX.

Analysis of plasma lipid metabolites revealed that glycerol was chronically elevated over the 4 weeks after UniNX, suggesting enhanced lipolysis after UniNX. Indeed, increased ATGL and HSL lipase mRNA levels were found in IWAT and EWAT. Although one may have expected that circulating triglycerides and fatty acids increased in plasma associated with the increased lipolysis, these were not observed, possibly because of increased lipid clearance. Indeed, fatty acid transporter CD36 mRNA was elevated in the liver and in selected tissues (kidney and gastrocnemius). This may explain, at least in part, the previously reported findings (Zhao et al., 2008, 2011) that UniNX led to excessive fatty infiltration and lipid accumulation in tissues (as determined by histology), albeit at time points greater than 3–6 months. Since we did not observe fatty infiltration in liver and kidney in our shorter duration study of 4 weeks, intracellular lipids are likely handled in a different manner in our time frame. They may be metabolized more rapidly, but not completely oxidized as is suggested by the higher circulating β-hydroxybutyrate and lack of increased UCP1 in brown adipose tissue.

Our data showed that circulating levels of hormones that regulate energy expenditure and body fat, such as leptin, T3, insulin, ghrelin, angiotensin 1 and angiotensin 2, were not significantly different between the two groups over the 4 week period. Hence, these hormones are unlikely to explain the activation of lipolysis (i.e., elevated circulating glycerol levels, increased fat pad lipases ATGL and HSL levels). Similar lack of differences in hormones have been found in UniNX in ad lib standard chow-fed rats in the first 6 months after UniNX (Zhao et al., 2011).

Other potential candidates for increasing lipolysis are cytokines. Of the increased circulating cytokines, IFNγ is of particular interest since its elevation induces both lipolysis and increases circulating ketone bodies *in vivo* (Khovidhunkit et al., 2004), which is what we observe in the UniNX group. Furthermore, our *in vivo* data reveal increased circulating neopterin and increased macrophage ROS production, which are both IFNγ-dependent. IFNγ has also been shown to increase lipid metabolism *in vitro* in adipocytes (Waite et al., 2001), kidney mesangial cell culture (Hao et al., 2013) and in whole animals studies (Feingold et al., 1992). We showed that other circulating cytokines known to induce lipolysis such as ASP, TNFα, and IL1α were also elevated. GM-CSF and erythropoietin have not been shown to directly mediate lipolysis but they can clearly regulate body weight and fat in rodent models (Reed et al., 2005; Lee et al., 2008; Meng et al., 2013; Alnaeeli et al., 2014).

Interestingly we showed that UniNX resulted in anti-inflammatory state in most tissues and this was associated with reduced cytokines in tissues such as liver, kidney and fat pads. Recently, it has been shown that in mouse UniNX models, tissues including fat pads showed a reduced inflammatory state (Sui et al., 2010; Chin et al., 2015). In our study in contrast, IFNγ and GM-CSF protein levels were increased in the UniNX spleen, suggesting that the increased circulating levels of these cytokines may arise from the spleen. A role for cytokine production by the spleen after kidney removal has been shown in mice (Andres-Hernando et al., 2012). Furthermore, nephrectomy can activate immune cells in the spleen (Lukacs-Kornek et al., 2008). In human kidney donors, the activation of cytokine signaling pathways through STATs and SOCS has been shown to occur (Xu et al., 2014).

Since we had previously shown increases in bile salts following UniNX, we chose to investigate whether bile salt receptor FXR (Penno et al., 2013; Gai et al., 2014a) and its potential regulator SIRT1 (Garcia-Rodriguez et al., 2014) were altered in various tissues. Here we show that both were modified in tissues by UniNX. These two factors can regulate not only metabolism but also inflammation. We observed an inverse relationship between tissue cytokine levels and tissue anti-inflammatory FXR/SIRT1 protein levels. The higher the tissue cytokines (in spleen), the lower the FXR/SIRT1 protein levels, and conversely the lower the cytokines (in adipose tissue, liver, kidney), the higher the FXR/SIRT1 levels. Although we have previously shown that the bile salt receptor FXR at the mRNA level showed a tendency to be elevated in the liver (Gai et al., 2014a), we now provide evidence that UniNX may increase FXR protein levels in liver, kidney and IWAT. Whether these increases represent the active form of the FXR warrants further studies. Interestingly SIRT1 may affect activity of various other signaling pathways by modifying the acetylated state of regulatory proteins including STATs (Liu et al., 2014). The age-dependent fatty infiltration of tissue could also potentially be attributed to decreased SIRT1, which is known to be down-regulated with age and is considered responsible for age-related metabolic changes (Kitada et al., 2013). It would therefore be of interest to determine whether these age effects of UniNX pathological fat infiltration and increased tissue inflammation are associated with decreases in SIRT1. The increases in FXR and SIRT1 levels found here in non-immune tissues also support our findings of the leaner phenotype following UniNX.

In summary, our study shows that, under conditions of a fixed intake of normal chow, young male rats that have undergone UniNX had lower body fat. This was associated with enhanced lipolysis and was paralleled by increases in subsets of circulating cytokines rather than changes in circulating hormones levels. Of the measured cytokines, IFNγ appear to be the best candidate for explaining body composition changes after UniNX, based on our *in vivo* physiological activation of IFNγ (increased circulating neopterin, β-hydroxybutyrate and increased macrophage ROS production). Further studies are required to determine whether these cytokines, and especially IFNγ, are acting directly on peripheral tissue or indirectly via the brain. Support for kidney–brain interactions have been shown to occur in chronic kidney disease induced by 5/6 nephrectomy (Mak et al., 2006; Cheung and Mak, 2012) and which results in wasting/cachexia. Altered body composition with loss of body fat and lean mass (Cheung et al., 2008; Cheung and Mak, 2012) after 5/6 nephrectomy implicates cytokines and central melanocortin 4 receptor (MC4R) pathways. However, the neuronal circuits involved, and whether these neurons have receptors for cytokines, remain to be demonstrated.

## Author contributions

Conceived and designed the experiments: JM, AD, DA. Performed the experiments: DA, JC. Analyzed the data: DA, JM, AD. Wrote paper: DA, JM, AD. Edited manuscript: DA, JM, AD.

### Conflict of interest statement

The authors declare that the research was conducted in the absence of any commercial or financial relationships that could be construed as a potential conflict of interest.

## Abbreviations

ASP: acylation stimulating protein
ATGL: adipose triglyceride lipase
FXR: farnesoid x receptor
GM-CSF: granulocyte-macrophage colony stimulating factor
HSL: hormone sensitive lipase
IFNγ: interferon-gamma
UniNX: uninephrectomy.
